# Postoperative Outcomes Following Rezum: A Novel Minimally Invasive Therapy for Benign Prostatic Hyperplasia (BPH)

**DOI:** 10.7759/cureus.90179

**Published:** 2025-08-15

**Authors:** Muhammad Raheel, Jaskarn Rai, Bilal Ahmad, Malek Gashaan, Roocha Odedra, Antoni J Bochinski

**Affiliations:** 1 Urology, Leicester General Hospital, University Hospitals of Leicester NHS Trust, Leicester, GBR; 2 Urology, Leicester Royal Infirmary, University Hospitals of Leicester NHS Trust, Leicester, GBR

**Keywords:** benign prostatic hyperplasia (bph), lower urinary tract symptoms (luts), postoperative outcome., rezum, urodynamic studies

## Abstract

Objectives: This study aimed to analyze and evaluate the effectiveness and safety of Rezum (water vaporization of the prostate), a minimally invasive treatment for managing lower urinary tract symptoms (LUTS) due to benign prostatic hyperplasia (BPH), with a focus on postoperative outcomes.

Methodology: Seventy-three patients meeting the inclusion criteria were enrolled in the study at Leicester General Hospital (University Hospitals of Leicester NHS (UHL), NHS trust), Leicester, England, from 1^st^ August 2023 to 31^st^ August 2024. In this prospective cohort study, the following variables were recorded: name, date of birth, date of admission, pre-/postoperative International Prostate Symptom Score (IPSS), pre-/postoperative Quality of Life Score (QOLS), pre-/postoperative International Index of Erectile Function (IIEF-5), prostate size, urodynamics, prostate-specific antigen (PSA) level, Rezum injections, anesthesia, post-procedure side effects and symptoms on follow-up, recommendations to family and friends, and status on discharge. To assess the procedure outcomes, these variables were analyzed using IBM SPSS Statistics, version 23 (IBM Corp., Armonk, NY), and the results were depicted in the form of graphs, charts, and tables.

Results: In our study, the mean preoperative IPSS was 22.6 ± 7.5, reduced to 12.8 ± 9.4, showing a 27% improvement. The highest pre-procedure QOLS was five (n=19, 26.4%), compared to three (n=21, 29.2%) after Rezum, representing a 33.3% improvement. Preoperative assessment of erectile function, as measured by the IIEF-5 with a maximum possible score of 25, revealed a mean score of 15.1 ± 1.1. Post-operative evaluation indicated a slight decline, with the mean score decreasing to 14.7 ± 4.03, reflecting a 1.5% reduction in erectile function. The reduction in symptomatic LUTS showed a statistically significant improvement with injections targeting the left side and the median lobe (p = 0.002). Furthermore, a statistically significant correlation was observed between Rezum injections targeting the median lobe and post-procedure quality of life (p = 0.003).

Conclusion: Through this single-center retrospective cohort, we concluded that Rezum is a minimally invasive outpatient procedure that offers safe and effective treatment for LUTS. It has a low side-effect profile and preserves sexual function. Further long-term studies are recommended.

## Introduction

Lower urinary tract symptoms (LUTS) have a considerable negative impact on the quality of life for men, especially for those aged 65 and older [1]. Various diseases of the genitourinary system have been recognized as causes of LUTS in men, potentially leading to acute urinary retention, reduced flow rates, and the potential requirement for surgery due to progressive prostate enlargement [2-3]. LUTS can be categorized as the voiding type (poor stream and hesitancy) and the storage type (increased frequency and urgency of passing urine) [4]. The primary reason for LUTS is the non-cancerous enlargement of the prostate, which impacts 40% of men in their 50s and 90% of men aged over 90 [5]. Benign prostatic hyperplasia (BPH) leads to bladder outflow obstruction (BOO) in 6% of cases of males globally [6]; hence, it is considered not only cumbersome and bothersome for the patient but also an economic burden on the healthcare system worldwide. 

Several treatment modalities have been utilized to manage BOO due to BPH. Conservative management in the form of pharmacological therapies (both monotherapy and in combination) [7] and lifestyle modifications (such as reduced fluid and caffeine intake) has been the initial mainstay of treatment for several decades [8]. When non-compliance and drug-related side effects [9] lead to treatment failure, invasive procedures, such as surgical removal or resection of the prostate gland, may be required. Transurethral resection of the prostate (TURP) has been the traditional surgical procedure for treating BPH in the modern era, indicated for moderate (eight to 19) to severe (20-35) LUTS not responding to medical treatment [10]. As of now, it remains the benchmark treatment for BPH despite having a significant adverse effect profile, which includes reoperation, urethral strictures, urinary tract infections, bleeding, incontinence, and sexual/erectile dysfunction [11-12]. 

For these reasons, urologists are introducing novel techniques related to minimally invasive surgery into their armamentarium to further cope with LUTS in better ways to improve the overall quality of life. Examples of these techniques include prostatic urethral lift (PUL), aqua-ablation, prostatic arterial embolization (PAE), and transurethral prostate convective radiofrequency water vapor therapy (Rezum) [13]. Approved by the FDA in 2015, Rezum, due to its performance in managing LUTS, has achieved great popularity among urologists throughout the world [14]. This procedure converts water into energized vapors by using radiofrequency and delivers the vapors into the prostatic tissue under cystoscopic control, leading to cellular destruction (coagulative necrosis) followed by gradual tissue resorption. Though its use is limited to relatively smaller prostates (<80 cc) [15], many centers are performing this procedure on an outpatient basis, and the minimal anesthesia requirements have further augmented its acceptance [16-17]. 

In our research, we sought to assess the clinical results of the initial group of patients who received treatment with the Rezum system at our university hospital, specifically focusing on postoperative outcomes. 

## Materials and methods

After taking the hospital's ethical and audit committee into confidence and maintaining all the rules and regulations related to conducting a clinical study, this analytical prospective cohort began in the Department of Urology at Leicester General Hospital (UHL), NHS Trust in Leicester, England. A total of 73 male patients aged above 40 years were enrolled using consecutive non-probability sampling.

The study duration was one year, and data from August 2023 to August 2024 were kept in perspective. Data were collected on a pre-designed proforma from patients through emails, which were transferred to a Microsoft Excel sheet (Microsoft Corp., Redmond, WA) for record keeping. Inclusion criteria were age > 40 years (relatively younger), patients who wanted to preserve their sexual function, patients with comorbidities making them unfit for prolonged anesthesia, and having BOO from BPH due to failure of conservative management. All other diseases related to the prostate, such as prostatitis, prostatic abscess, and prostatic cancer, and younger patients were excluded.

The variables that were included in this research are the following: name, date of birth, date of admission, pre-/postoperative International Prostate Symptom Score (IPSS), pre-/postoperative Quality of Life Score (QOLS), pre-/postoperative International Index of Erectile Function (IIEF-5), prostate size, urodynamics, prostate-specific antigen (PSA) level, Rezum injections, anesthesia, post-procedure side effects and symptoms on follow-up (judged with the help of ACCURX software (Accurx, London, United Kingdom) within a minimum period of three months), recommendations to family and friends, and status on discharge. Quantitative variables such as IPSS, QOLS, IIEF, and number of injections were analyzed as mean ± standard deviation, and qualitative variables were analyzed as frequency and percentages. The post-stratification chi-square test was utilized to measure the strength of association between the variable and outcome, keeping the p-value at < 0.05 as statistically significant. The Rezum system was used following the manufacturer’s recommendations for the treatment of both prostatic lobes as well as the central zone or median lobe as described in the literature [18]. Each participant in the study gave their written informed consent for the use of their clinical data in medical research analysis. The data was entered from proformas into a Microsoft Excel sheet and then transferred to IBM SPSS Statistics software, version 23 (IBM Corp., Armonk, NY) for analysis. The results were depicted in the form of graphs, tables, and charts.

## Results

**Figure 1:**
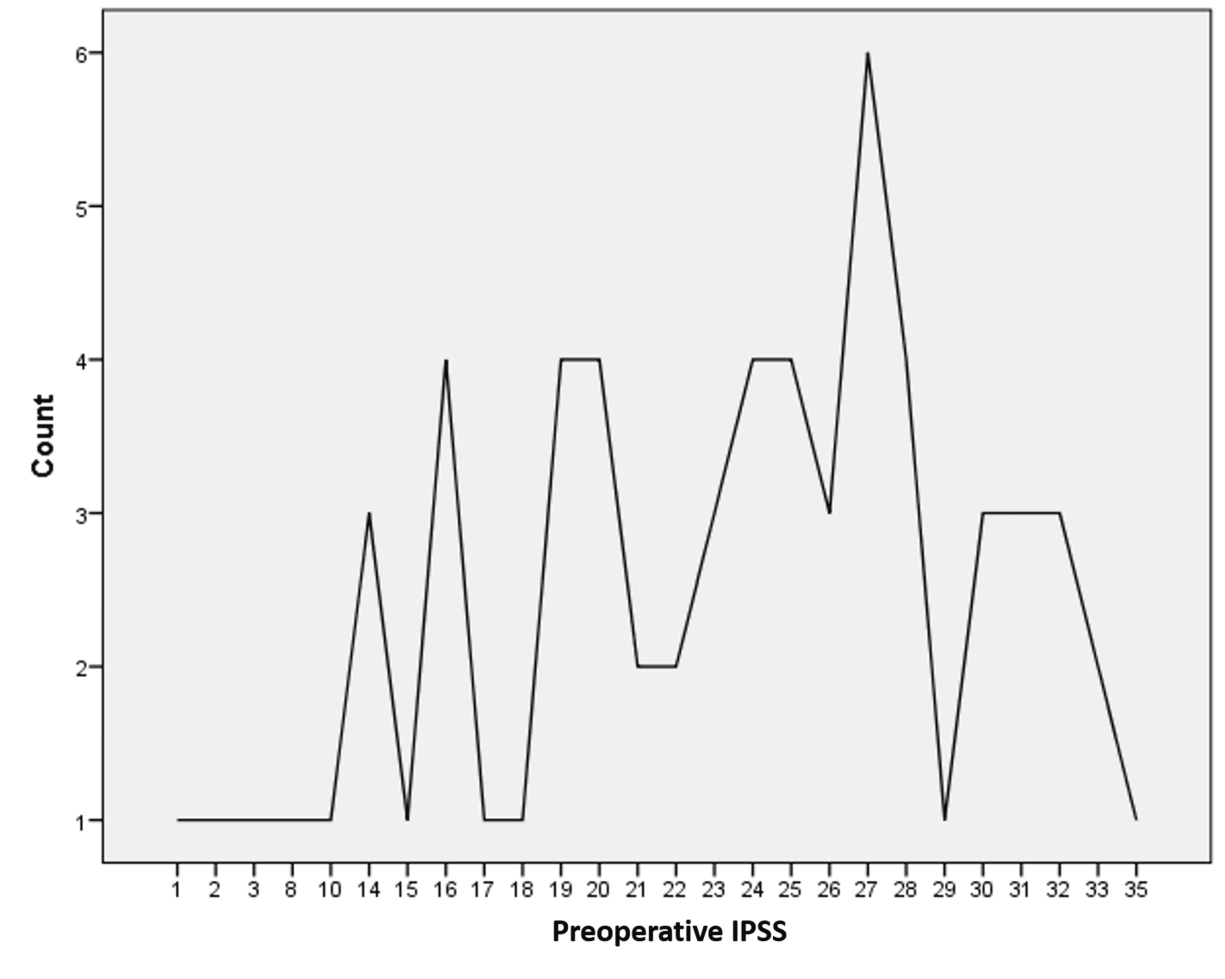
Frequencies for various counts of preoperative International Prostate Symptom Score (IPSS)

Of the 73 patients enrolled, 72 completed the study. The mean age of presentation was 69.5 ± 1.2 years, and the mean PSA level obtained was 2.71 ± 0.34 ng/mL. The mean preoperative IPSS was 22.6 ± 7.5 (range 1-35), improving to 12.8 ± 9.4 postoperatively (maximum = 28 and minimum = 0) (p < 0.05). Full details are shown in Figure 1 and Table 1.

**Table 1:** Variables with their demographics PSA: prostate-specific antigen; QOLS: Quality of Life Score; IPSS: International Prostate Symptom Score; IIEF: International Index of Erectile Function

Variable	Mean	Standard deviation	Maximum	Minimum
Age (years)	69.5	1.2	92	46
PSA (ng/mL)	2.71	0.34	9.4	0.1
Preoperative IPSS	22.6	7.5	35	1
Postoperative IPSS	12.8	9.4	28	0
Preoperative QOLS	4.36	1.6	5	1
Postoperative QOLS	2.3	0.15	4	0
Preoperative IIEF	15.1	1.1	25	0
Postoperative IIEF	14.7	4.03	19	0

**Figure 2:**
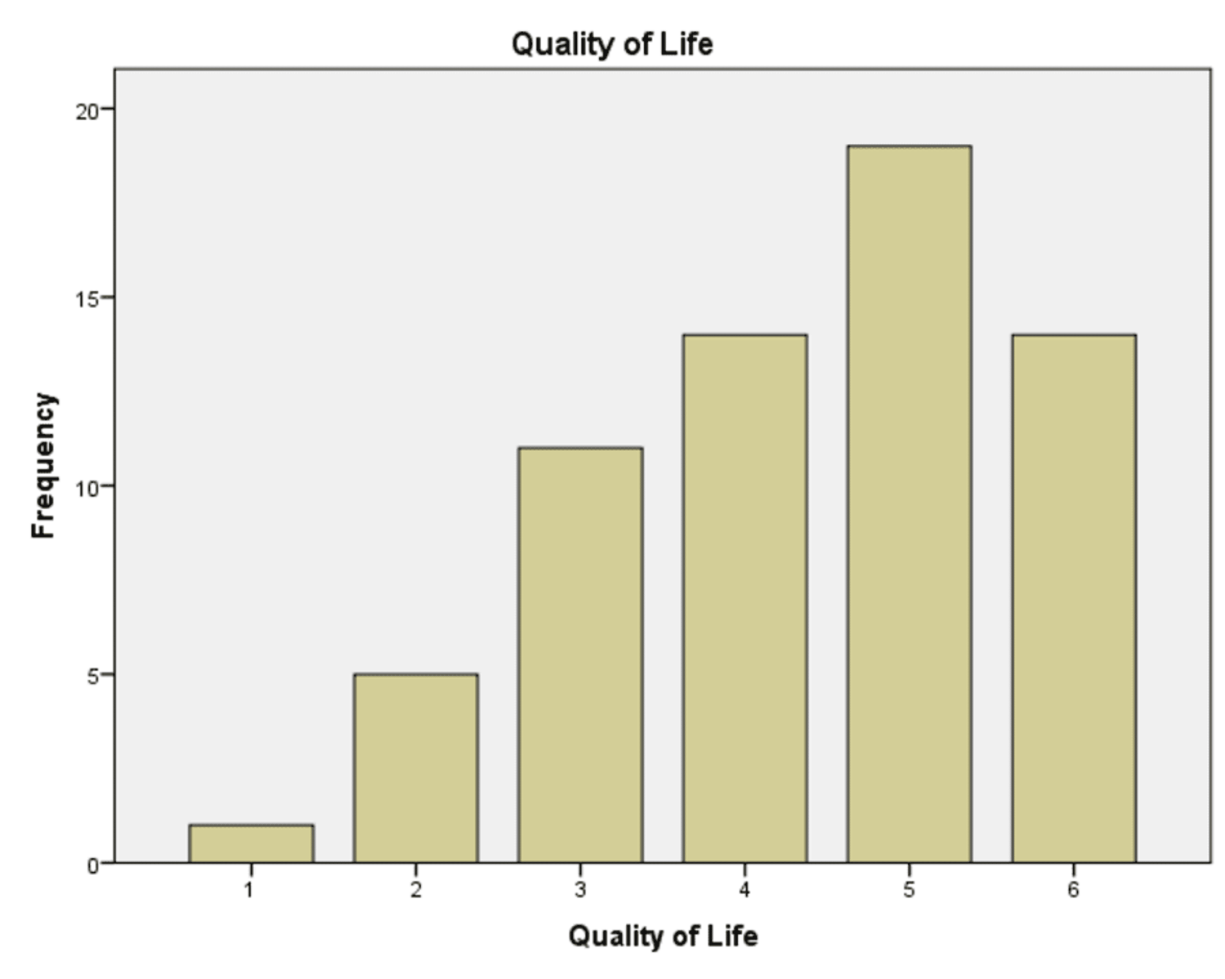
Pre-Rezum Quality of life Score (QOLS)

**Figure 3:**
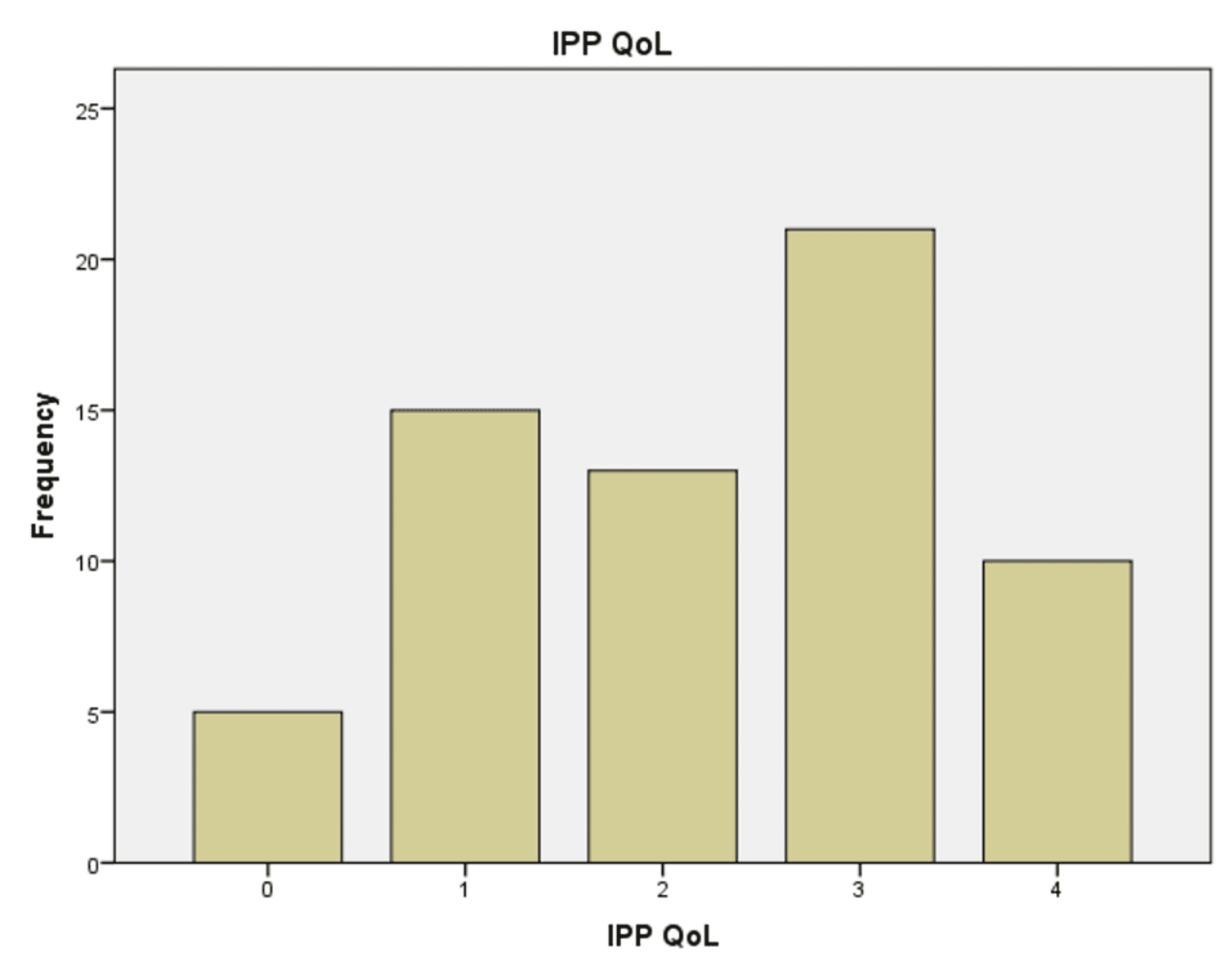
Post-Rezum intravesical prostatic protrusion (IPP) measured against the Quality of Life Score (QOLS)

The pre-procedure QOLS was also utilized to evaluate the outcomes, as shown in Table 1 and Figure 2, with the highest number of patients at score five (n=19, 26.4%) and the lowest score at one (n=1, 1.4%) compared to post Rezum (Figure 3), with the highest number at three (n=21, 29.2%) and the lowest score at 0 (n=5, 6.9%), hence exhibiting a 33.3% improvement. QOLS, according to IPSS, was classified as follows [19]: "terrible" (6), "unhappy" (5), "mostly dissatisfied" (4), "mixed" (3), "mostly satisfied" (2), "pleased" (1), and "delighted" (0). 

**Figure 4:**
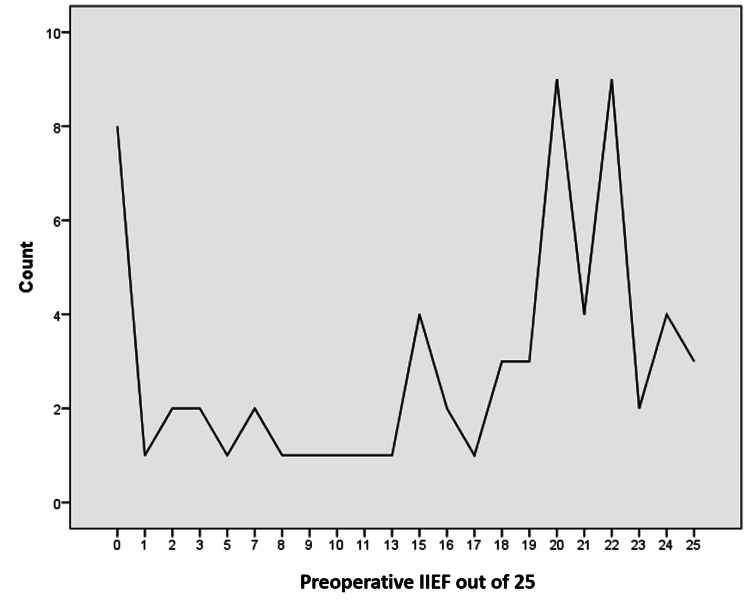
Pre-Rezum International Index of Erectile Function Score (IIEF)

The IIEF is a survey designed to evaluate erectile dysfunction and its level of severity. The IIEF scores ranged between five and 25, categorizing ED into five levels: severe (five to seven), moderate (eight to 11), mild to moderate (12 to 16), mild (17 to 21), and no ED (22-25) [20]. Preoperative documentation of IIEF-5 scores revealed a mean score of 15.1 ± 1.1 (maximum=25 and minimum=5), while post-procedure scores showed a mean of 14.7 ± 4.03 (maximum=19 and minimum=5), exhibiting a decline of 1.5%. Complete details are depicted in Figure 4 and Table 1. 

Study patients received injections on the left, right, and median lobes. An average of 2.54 left-sided injections with a maximum of six and a minimum of one were administered. The maximum number of patients required two injections (n=38, 52.8%), and the remaining patients had six injections (n=2, 2.8%). Similarly, for the right side, an average of 2.54 injections was utilized. The maximum number of patients required two injections (n=35, 48.6%), and the remaining patients had six injections (n=1, 1.4%). For the median lobe, having an average of 0.93 injections, the maximum number of candidates did not receive any injection at all (n=29, 40.3%), and the least number of patients had four injections (n=1, 1.4%). In terms of anesthesia, general anesthesia (GA) was given to 42 patients (58.3%), spinal anesthesia to two patients (2.8%), and local anesthesia to 27 patients (37.5%). A hospital-based application (ACCURX) was employed to gather information from the patients on follow-up, through which 47 (65.3%) patients responded, while the remaining 25 patients (34.7%) were contacted directly via telephone or email due to non-response on ACCURX. Among all respondents, 31 patients reported postoperative side effects (43.1%), while 34 (47.2%) did not. 30 patients (41.6%) sought healthcare assistance for issues such as nocturia, orchitis, dysuria, urgency, urinary retention, and blocked catheters. Symptomatic improvement in LUTS was reported by 49 patients (68.1%). Twenty-eight patients were satisfied on discharge (38.8%), and 52 patients agreed to recommend this procedure to their family/friends (72.2%). Further demographic details are given in Table 2. 

**Table 2:** Postoperative outcomes and patient feedback LUTS: lower urinary tract symptoms

Variable	Frequency	Percentage
Anesthesia		
General anesthesia	43	59.7
Spinal	2	2.8
Local	27	37.5
ACCURX follow-up		
Yes	47	65.3
No	25	34.7
Postoperative side effects		
Yes	31	43.1
No	34	47.2
No record available	5	6.9
Visited healthcare professionals since the procedure		
Yes	30	41.6
No	36	50
No record available	6	8.3
Symptoms improved (LUTS)		
Yes	49	68
No	17	23.6
No record available	6	8.3
Recommendations to family/friends		
Yes	52	72.2
No	15	20.8
No record available	5	6.9
Happy to be discharged		
Yes	28	38.8
No	39	54.2
No record available	5	6.9

The Pearson chi-square test was employed to evaluate the association between the procedure (Rezum) and postoperative outcomes, including IPSS, QOLS, IIEF, and symptom improvement. A statistically significant improvement in symptomatic LUTS was observed with left-sided and median lobe injections (p-value = 0.002). Moreover, Rezum injections targeting the median lobe showed a significant correlation with post-procedure quality of life (p-value = 0.003). Erectile dysfunction improvement was seen following left-sided Rezum injections (p-value = 0.003). Further details are provided in Table 3. 

**Table 3:** Correlation of injections with post-procedure outcomes QOLS: Quality of Life Score; IPSS: International Prostate Symptom Score; IIEF: International Index of Erectile Function; LUTS: lower urinary tract symptoms

Variable	Post-procedure outcome	P-value
Injections on the left side	Postoperative IPSS	0.790
	Postoperative QOLS	0.361
	Postoperative IIEF	0.003
	Postoperative side effects	0.206
	Symptom improvement (LUTS)	0.002
Injections on the right side	Postoperative IPSS	0.973
	Postoperative QOLS	0.239
	Postoperative IIEF	0.034
	Postoperative side effects	0.110
	Symptom improvement (LUTS)	0.566
Median lobe	Postoperative IPSS	0.003
	Postoperative QOLS	0.923
	Postoperative IIEF	0.146
	Postoperative side effects	0.951
	Symptom improvement (LUTS)	0.002

## Discussion

We are living in an era of continuous advancement in the medical field, and it is encouraging to witness a ceaseless trend in clinical trials worldwide to decrease patient morbidity following invasive procedures. A minimally invasive surgical procedure can be characterized as one that ensures safety and results in reduced postoperative complications for patients when compared to a traditional method for the same surgery. Interestingly, the initial procedure that avoided a prior radical surgery was the application of a cystoscope to examine and address bladder lesions [21]. This research aimed to evaluate the efficacy of water vapor thermal therapy for the prostate, known as the Rezum system, in a sequential group of patients. 

Several factors were evaluated to determine the durability of this new minimally invasive approach for treating LUTS resulting from BPH. In our research, the average age at presentation was 69.5 ± 1.2 years, and the average PSA level recorded was 2.71 ± 0.34. The mean preoperative IPSS was 22.6 ± 7.5, which was reduced to 12.8 ± 9.4, showing a 27% improvement. The highest pre-procedure QOLS was five (n=19, 26.4%), compared to three (n=21, 29.2%) after REZUM, representing a 33.3% improvement. Regarding managing LUTS, 49 patients disclosed improvement in LUTS (68.1%), 28 patients were satisfied with discharge (38.9%), and 52 patients agreed to recommend this procedure to their family/friends (72.2%). This trend has been observed in previous trials. Wolters et al. [22] illustrated that there were improvements of about 50% in both IPSS and IPSS-QoL scores, while McVary et al. [23] reported a 46.7% decrease in IPSS and a 42.9% decrease in IPSS-QoL in their study. Cindolo et al. [24] reported the case of a 50-year-old male patient with LUTS related to BPH (IPSS 21, QoL four) who had an improvement in his symptoms (IPSS four, QoL one) at a two-month follow-up. 

In many surgical procedures for BPH, a significant occurrence of adverse effects on ejaculatory function is frequently observed. This group also assessed sexual function by employing the IIEF-5. Preoperative documentation of the IIEF showed a mean score of 15.1 ± 1.1, which decreased to a mean score of 14.7 ± 4.03 postoperatively, indicating a minor decline in erectile function of 1.5%. The Rezum procedure works by delivering water vapor to the prostate tissue, which can cause some damage to the nerves involved in ejaculation, potentially leading to altered ejaculatory function. These findings contradict the results of Zhu et al. [25] and Westwood et al. [8], who reported no de novo erectile dysfunction but rather a preservation of sexual function, while three studies reported ejaculatory dysfunction rates between 2.4 and 3.1% [26]. Emerging evidence indicates that with optimized procedural techniques and postoperative care, erectile function can be preserved or even improved following Rezum therapy. To enhance outcomes, it is crucial to preserve neurovascular integrity during the procedure, possibly by utilizing advanced imaging and intraoperative nerve monitoring. Selecting patients without severe baseline erectile dysfunction ensures that postoperative results accurately reflect the procedure’s impact. Longer follow-up periods are essential, as nerve recovery may lead to improved erectile function over time. Implementing structured postoperative rehabilitation, including pharmacotherapy (e.g., PDE5 inhibitors) or lifestyle interventions, can further support recovery. Future studies should analyze the effects of different injection sites and techniques on IIEF outcomes to refine procedures and minimize adverse effects. Conducting multicenter trials would provide more representative data to validate these findings and refine protocols. 

In addition, some minor adverse events in the form of urinary tract infections, urgency, nocturia, dysuria, and blocked catheters were also seen, supporting the findings of Dixon et al. [27]. A total of 11 patients (15.3%) experienced urinary tract infections following surgery (Clavien-Dindo grade 2), necessitating a course of oral antibiotics for two weeks. Acute urinary retention (AUR) occurred in six patients (8.3%) following the removal of the catheter, for whom re-catheterization was performed, compared to Tuna et al. [28], in their study, where nine patients had AUR. 

In our cohort, the average number of injections received was five (two on the left, two on the right, and one on the median lobe), similar to the median number of six injections in the study by Tuna et al. [28] and to an average of eight injections in the study by Noureldin et al. [29], as opposed to 21 injections in a 65-year-old male patient in the study by Cindolo et al. [24]. Two patients with preoperative indwelling long-term catheters (LTC) were catheter-free following the procedure in our study. Various authors have published their ideas regarding the optimum time for catheter removal. We believe it's related to patient factors such as previous injuries, retentions, incontinence, and the size of the prostate. Johnston et al. [30] indicated that the typical duration for catheter removal is between three and five days; however, for patients with a history of urinary retention and larger prostates, the catheters were retained for an extended period. Mostly general anesthesia was administered to patients in our study (n=43, 59.7%); however, there are advocates of using local anesthesia during Rezum due to less operative time [31, 32]. In the future, encouraging the implementation of regional anesthesia techniques would be beneficial. 

Some limitations of this clinical trial are important to consider. It is important to note the small sample size and single-center design, along with the limited follow-up period. To evaluate the impact of Rezum on patient outcomes, post-procedure urodynamic measurements such as flow rate, maximum flow rate (Qmax), post-void residual volume (PVR), BOO, and equilateral obstruction (Eq Ob), as well as prostate size, would provide a better understanding of the relevance of this method in our context. 

## Conclusions

The Rezum procedure (water vapor therapy) is a safe and effective treatment for managing symptoms of BPH with positive results in the short-term follow-up. The primary benefits include its favorable safety profile, promising midterm outcomes, and excellent maintenance of sexual function, establishing it as a flexible option in urology. It is an encouraging novel approach that can also be applied to patients with median lobe disorders. Although there is a slight risk of temporary, minor complications, it can be utilized in an outpatient environment as well. Further investigation is necessary to explore the potential to expand the inclusion criteria for Rezum. 
